# Internal Model-Based Robust Tracking Control Design for the MEMS Electromagnetic Micromirror

**DOI:** 10.3390/s17061215

**Published:** 2017-05-26

**Authors:** Jiazheng Tan, Weijie Sun, John T. W. Yeow

**Affiliations:** 1 College of Automation Science and Engineering, South China University of Technology, Guangzhou 510000, China; 201520112765@mail.scut.edu.cn (J.T.); auwjsun@scut.edu.cn (W.S.); 2 Department of Systems Design Engineering, University of Waterloo, Waterloo, ON N2L 3G1, Canada

**Keywords:** internal model, robust tracking, output regulation problem, micromirror

## Abstract

The micromirror based on micro-electro-mechanical systems (MEMS) technology is widely employed in different areas, such as scanning, imaging and optical switching. This paper studies the MEMS electromagnetic micromirror for scanning or imaging application. In these application scenarios, the micromirror is required to track the command sinusoidal signal, which can be converted to an output regulation problem theoretically. In this paper, based on the internal model principle, the output regulation problem is solved by designing a robust controller that is able to force the micromirror to track the command signal accurately. The proposed controller relies little on the accuracy of the model. Further, the proposed controller is implemented, and its effectiveness is examined by experiments. The experimental results demonstrate that the performance of the proposed controller is satisfying.

## 1. Introduction

In recent years, many engineers, as well as scientists have devoted themselves to the research of MEMS technology because of its outstanding potential in the field of fundamental research, as well as industrial application. Among all of the research works of MEMS technology, optical MEMS is a fashionable one, and especially the torsional micromirror is one kind of very representative optical MEMS device. The torsional micromirror has an extensive range of applications, such as optical coherence topography [1], optical switches [2], high resolution displays [3], and so on. Based on the actuation method, the torsional micromirrors can be classified into different types, such as electrothermal [4], electrostatic [5], electromagnetic [6,7,8] and piezoelectric [9]. All of these different kinds of micromirrors have their own characteristics, which are shown briefly as follows. The electrothermal micromirror guarantees the advantage of a large deflection angle, but it needs large energy consumption and has a long response time, which is suitable for the low frequency scanning application [10]. In contrast, the piezoelectric micromirror needs low energy consumption and has a fast response, but its range of deflection is small due to the material restriction, which is applicable for high speed scanning [11]. As for the case of the electrostatic micromirror, it features superiority in its fast response and large deflection angle. However, its energy consumption is considerable, and the so-called pull-in phenomenon exists, which bothers engineers deeply. Therefore, one of its suitable uses is optical switches [12]. Compared with these three kinds of micromirrors, the electromagnetic micromirror obtains superiority, such as producing large scan angles with low actuating voltage, low energy consumption and remote actuation [6]. Be aware that, to construct a device based on a electromagnetic micromirror, the peripheral drive circuit based on microcoils is essential, leading to the difficulty in manufacturing. However, in recent years, the development of an integrated technique makes it much easier to overcome this difficulty. Thus, gradually, the electromagnetic micromirror attracts more and more attention. Due to the above superiority, this paper focuses on the electromagnetic micromirror.

In addition to the development of manufacturing technology, designing the controller for the micromirror to solve a specific problem is another great way to improve the performance. Some examples are provided as follows. The nonlinear proportion-integral-derivative (PID) controller [13] and the nonlinear proportion-derivative (PD) [14] controller are designed for the electrostatic micromirror to improve the transient performance. In [15], the sliding mode controller is applied to improve the electrostatic micromirror’s robustness. As for the electrothermal micromirror, the linear-quadratic-Gaussian (LQG) control is used to improve the anti-noise ability [16]. In [17], the feedforward control method is applied to overcome the hysteresis problem for the piezoelectric micromirror.

The aim of our paper is to study the tracking control problem for the electromagnetic micromirror by designing an effective controller. Our research motivation is shown as follows. Firstly, the above examples illustrate clearly that the control design is an effective way to help solve a specific problem for the MEMS micromirror and is much more low cost than the improvement of MEMS fabrication technology. Secondly, because of the aforementioned advantages, the electromagnetic micromirror has real potential to be widely applied to various kinds of scanning or imaging applications. To suit these actual requirements, the electromagnetic micromirror usually needs to track the given reference, and some similar examples [18,19] indicate that robust and precise tracking performance is essential in some of the application scenarios. For instance, the micromirror is required to track the sinusoidal reference to meet the demand of the bar code scanning application.

In control theory, the above issues can be formulated as an output regulation problem, which we are going to give a brief overview of as follows. The core of the output regulation problem is to design a controller such that, in addition to keeping the closed-loop system stable, the output is able to achieve zero tracking errors of the reference trajectories. The problem has been studied for a long time because of its academic and practical interests. For a linear system, it has gained convincing research results [20,21,22,23]. Recently, the two main issues of the output regulation problem are the case of nonlinear systems, as well as the case of multi-agent systems. Both topics have achieved many results [24,25,26,27,28]. However, up till now, there are still many interesting topics in this area that remain open. As we will introduce in Section 2, the model of the electromagnetic micromirror is linear. Thus, we are focusing on the theory of the linear output regulation problem. In the framework of the linear output regulation problem, two main candidates can be chosen to solve the problem, the feedforward-based approach and the internal model-based approach. For the first choice, it sets a great demand on the model accuracy of the given object [23]. However in practice, there always exists model uncertainty, particularly in microcosmic system applications, such as MEMS fields. Clearly, this choice is hardly worth serious consideration in practice. Conversely, the internal model-based controller can withstand model uncertainty within a range and maintain precise tracking performance at the same time, which is able to solve the tracking control problem robustly. Thus, such a method is much more suitable for actual applications.

Since the advantage of the internal model-based approach is apparent and fits the practical requirements satisfactorily, we design the tracking controller for the electromagnetic micromirror based on this design philosophy. The experimental results manifest the effectiveness of the proposed internal model-based controller clearly. Besides, for comparison, we design a well-known proportion-integral-derivative (PID) controller for the electromagnetic micromirror, and the effectiveness of PID is also provided. Contrasting the performances of the proposed controller with the PIDs, we are able to see the difference clearly, which further manifests the effectiveness of the proposed controller.

We organize the rest of the paper as follows. Section 2 provides a brief overview of the electromagnetic micromirror. In Section 3, the internal model-based controller is designed to solve the tracking control problem for the MEMS micromirror. Section 4 provides the simulation results of the proposed controller and the comparison with the PID control. In Section 5, we implement the proposed controller, and the effectiveness is verified. Finally, Section 4 concludes this paper.

## 2. Overview of the Electromagnetic Micromirror

In this section, the fabrication process of the electromagnetic micromirror is proposed in brief [6], and the dynamic model of the electromagnetic micromirror is shown. At the beginning, some essential instructions are given. The electromagnetic micromirrors can be classified according to the magnetic medium added into the micromirror, which are hard-magnetic and soft-magnetic. For a soft-magnetic micromirror, the deflection direction is unidirectional, and this sets a severe limitation of its application fields. Compared with the soft-magnetic micromirror, the hard-magnetic micromirror is able to deflect bidirectionally [6], which is clearly more practical. In this paper, the object we study is a hard-magnetic one.

Now, we are going to propose the fabrication process [6]. In the first place, adopting the lithography method, the mould of the micromirror and two torsional bars are fabricated with a silicon wafer used as the mould substrate. The mould is fabricated by standard photolithography. Next, polydimethylsiloxane (PDMS) is filled into the mould as a basement of the micromirror and two torsional bars, and the MQFP-12-5 isotropic magnetic powder (Nd-Fe-B) (Magnequench International Inc., Pendleton, IN, USA) with a particle size D50 of 5μm is doped into the polydimethylsiloxane (PDMS) at the weight percentage of 80%. Subsequently, an ultrasonic horn tip probe is immersed into the composite, therefore leading to uniform dispersion. Then, using the e-beam evaporation approach, the micromirror is plated with a layer of gold with a thickness of 1.0 mm as a reflective layer. Finally, the micromirrors are magnetized under a field of 1.8 Tesla, resulting in that the micromirror contains a hard magnetic feature. Besides, the rectangle coils are fabricated by the standard printed circuit board (PCB) manufacturing technique and constructed under the micromirror. The detailed design parameters of the mirror and coils are shown in Table 1 and Table 2, respectively. For any further details of the current micromirror, one can refer to them from [6].

It is remarkable that the aforesaid processing technology will lead to the surface asperities of the electromagnetic micromirror, which is against the scanning, as well as the imaging application. To overcome this problem, a reflective film is attached to the surface of the electromagnetic micromirror, resulting in the smoothness of its surface. Additionally, further dynamic tests illustrate clearly that this modifying method affects the capability of the micromirror little, which is proven essentially feasible.

**Figure 1:**
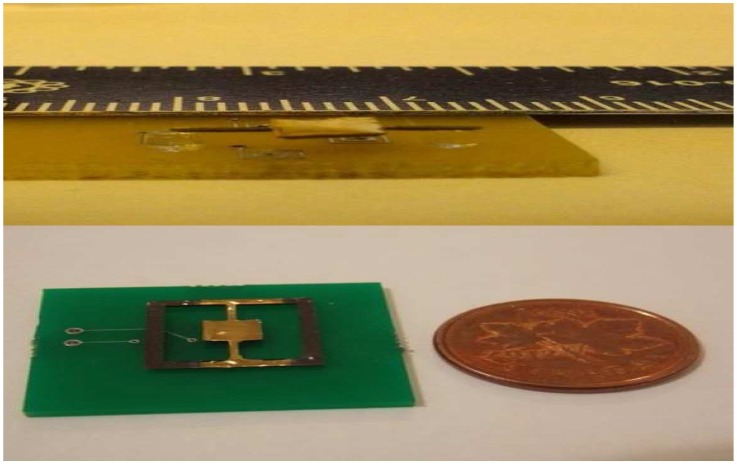
The structure of the micromirror.

The dynamic deflection model of the micromirror can be written as follows [7]:

(1) $$J\ddot{\theta }+b\dot{\theta }+k\theta =T\left(\theta \right)$$

where $\theta \in [-\frac{\pi }{2},\frac{\pi }{2}]$ denotes the angular deflection, *J* is the moment of inertia, *b* is the damping coefficient, *k* is the spring coefficient of the torsional bars and *T* denotes the driving magnetic torque produced by the Lorentz forces. Figure 1 shows the structure of the micromirror. As the current passes through the planar microcoils under the micromirror, the microcoils will be powered to generate the torque *T*, resulting in the angular deflection of the micromirror.

The moment of inertia is given as [7]:

(2) $$J=\frac{\rho_{m}W_{m}t_{m}{L_{m}}^{3}}{12}$$

and the spring coefficient can be written by [29]:

(3) $$k=\frac{2K_{ab}}{L_{beam}}$$

In the above equation, the cross-section shape-dependent factor $K_{ab}$ is expressed as [29]:

(4) $$\begin{array}{ccc} K_{ab} & = & \frac{GW_{beam}{t_{beam}}^{3}}{16}\left[5.33-3.36\frac{t_{beam}}{\alpha W_{beam}}\left(1-\frac{{t_{beam}}^{4}}{12\alpha^{4}{W_{beam}}^{4}}\right)\right] \end{array}$$

where α is the elastic deformation of the material anisotropy constant that is equal to one for the isotropic material, and *G* is the shear modulus of polydimethylsiloxane (PDMS) matrix:

(5) $$G=\frac{E}{2(1+v)}$$

with *v* the Poisson’s ratio of PDMS.

Additionally, the damping coefficient *b* can be calculated [30]:

(6) $$b=\frac{\sqrt{Jk}}{Q}$$

where *Q* is the quality factor of the micromirror that is approximately five.

Furthermore, the driving magnetic torque *T* is defined [30]:

(7) T(θ)=VMHcos(θ)

where *V* is the volume of the magnet, *M* is the saturation magnetization and *H* is the external magnetic field generated by the driving current *I*.

Notice that theoretically, the torque *T* is regraded as the control input of Equation (1). Nevertheless, notice that the driving current *I*, which is the real source of the external magnetic field *H*, is the practical control input variable that we can handle directly. Consequently, some necessary transformation needs to be established. Firstly, from Equation (7), it is obvious that the angular deflection has an impact on *T*. Because θ is able to be directly measured, based on Equation (7), we can obtain an input-output linearization law [31], which is shown as follows.

(8) $$u=\left\{\begin{array}{cc} \frac{T}{cos(\theta )}, & \theta \in (-\frac{\pi }{2},\frac{\pi }{2})\\ 0, & \theta =\pm \frac{\pi }{2} \end{array}\right.$$

Therefore, we obtain:

(9) $$u=\left\{\begin{array}{cc} VMH, & \theta \in (-\frac{\pi }{2},\frac{\pi }{2})\\ 0, & \theta =\pm \frac{\pi }{2} \end{array}\right.$$

Then, from Equation (9), we know that there exists a direct relationship between current *u* and torque *H*. Since *H* is related to *I*, we would figure out the relationship between *u* and *I* in the following. For this reason, a finite element analysis (FEA) simulation study of this relationship is given, which is shown in Table 3.

In summary, the essential transformation from *u* to *I* can be set in the following design process based on the above-mentioned analysis. Thus, we are able to continue our control input design directly based on the following differential equation:

(10) $$\begin{array}{c} J\ddot{\theta }+b\dot{\theta }+k\theta =u \end{array}$$

Define $x_{1}=\theta$ and $x_{2}=\dot{\theta }$. Then, we can obtain the state-space model of Equation (10):

(11) $$\begin{array}{ccc} \left[\begin{array}{c} {\dot{x}}_{1}\\ {\dot{x}}_{2} \end{array}\right] & = & \left[\begin{array}{cc} 0 & 1\\ a_{1} & a_{2} \end{array}\right]\left[\begin{array}{c} x_{1}\\ x_{2} \end{array}\right]+\left[\begin{array}{c} 0\\ b_{1} \end{array}\right]u\\ y & = & \left[\begin{array}{cc} 1 & 0 \end{array}\right]\left[\begin{array}{c} x_{1}\\ x_{2} \end{array}\right] \end{array}$$

where $a_{1}=-\frac{k}{J}$, $a_{2}=-\frac{b}{J}$ and $b_{1}=\frac{1}{J}$. In the following section, for convenience, we will express Equation (11) in a concise way, which is shown as below:

(12) $$\begin{array}{c} \dot{x}=Ax+Bu\\ y=Cx \end{array}$$

by defining $x={\left[\begin{array}{cc} x_{1} & x_{2} \end{array}\right]}^{T}$ and:

$$A=\left[\begin{array}{cc} 0 & 1\\ a_{1} & a_{2} \end{array}\right],\;B=\left[\begin{array}{c} 0\\ b_{1} \end{array}\right],\;C=\left[\begin{array}{cc} 1 & 0 \end{array}\right].$$

## 3. Design of a Internal Model-Based Controller

In this section, a internal model-based controller is designed for the micromirror, such that the micromirror can achieve a trajectory tracking of a desired reference F(t).

At the beginning, it is essential to demonstrate the internal model principle briefly. The internal model principle illustrates that if a closed-loop system incorporates in the feedback path a suitably reduplicated model of the dynamic structure of the reference, it can track the reference robustly [21]. This suitably reduplicated model in the feedback path is the so-called internal model. The existence of the internal model is able to ensure that the output of the closed-loop system tracks the reference robustly so long as the closed-loop system is stable.

In the following, we design the controller step by step. Firstly, we will do a necessary formulation to present the dynamic structure of the reference. To act as a scanner, the desired trajectory can be set as $F\left(t\right)=A_{m}\sin\left(\sigma t+\phi \right)$. F(t) can be expressed as an exosystem in the form of the state space equation. Define:

(13) $$\begin{array}{c} \dot{v}=A_{1}v \end{array}$$

Because $F\left(t\right)=A_{m}\sin\left(\sigma t+\phi \right)$, we obtain that $v=\left[\begin{array}{c} v_{1}\\ v_{2} \end{array}\right]$, $A_{1}\left(\sigma \right)=\left[\begin{array}{cc} 0 & \sigma \\ -\sigma & 0 \end{array}\right]$, $F\left(t\right)=v_{1}\left(t\right)$ with $A_{m}=\sqrt{v_{1}^{2}\left(0\right)+v_{2}^{2}\left(0\right)}$ and $\phi =\arctan\frac{v_{1}\left(0\right)}{v_{2}\left(0\right)}$. Furthermore, let $\Omega =(a_{1},a_{2},b_{1})$ and $\bar{\Omega }=\left({\bar{a}}_{1},{\bar{a}}_{2},{\bar{b}}_{1}\right)$ be the actual and nominal values of the system parameter vector, respectively. Then, $\Omega =\bar{\Omega }+w$ for some $w\in W\subset R^{3}$. Apparently, *w* represents the parameter uncertainty from its nominal value.

In this respect, the tracking control problem can be viewed as an output regulation problem that regulates the error output *e* of the following system:

(14) $$\begin{array}{c} \dot{x}=Ax+Bu\\ y=Cx\\ \dot{v}=A_{1}\left(\sigma \right)v\\ e=y-v_{1} \end{array}$$

to the origin asymptotically, i.e., $\lim_{t\to \infty }e\left(t\right)=0$, in the presence of parameter uncertainty *w*.

Then, based on the internal model principle, the solution to the output regulation problem is proposed without proving [23]. We can solve the output regulation problem of Equation (14) by using the following feedback controller:

(15) $$\begin{array}{c} u=K_{1}x+K_{2}z\\ \dot{z}=G_{1}z+G_{2}e,\;z={\left[\begin{array}{cc} z_{1} & z_{2} \end{array}\right]}^{T}\\ G_{1}=\Psi \left[\begin{array}{cc} 0 & 1\\ -\sigma^{2} & 0 \end{array}\right]\Psi^{-1}\\ G_{2}=\Psi {\left[\begin{array}{cc} 0 & 1 \end{array}\right]}^{T} \end{array}$$

where Ψ denotes any appropriate invertible two-dimensional matrix. For simplicity, we select Ψ as a two-dimensional identity matrix.

For convenience, we denote:

(16) $$\begin{array}{c} X=\left[\begin{array}{c} x\\ z \end{array}\right] \end{array}$$

Then, we can rewrite the whole system composed of Equations (14) and (15):

(17) x˙z˙=A+BK1BK2G2CG1xz+b0−1v1 =ACX+B0v1,b0=[000]T  y=C0.xz  v˙=A1(σ)v  e=y−v1

**Figure 2:**
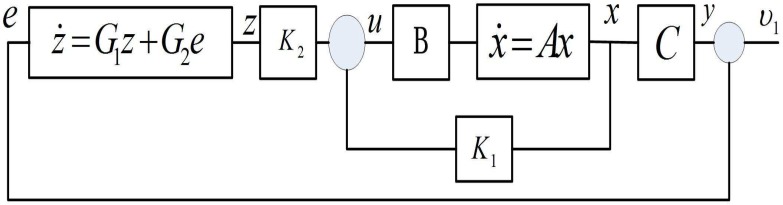
Block diagram of the proposed control design.

Clearly, the tracking control problem is solved through solving the output regulation problem. The output regulation problem can be tackled as long as $K_{1}$, $K_{2}$ is able to stabilize the above matrix $A_{C}$. The whole framework of the control design is shown in Figure 2. The proof of the stability analysis is omitted, and one can refer to them from [20,23].

In Equation (15),

(18) $$\begin{array}{c} \dot{z}=G_{1}z+G_{2}e \end{array}$$

is called an internal model of the closed-loop system composed of Equations (14) and (15). According to the internal model principle, it acts as a dynamic compensator to create a model of the dynamic structure of the given reference. Because of this, the form of $G_{1}$ and $G_{2}$ depends on the form of the given reference F(t).

One may suspect that the effectiveness of Equation (15) relies on the parameter accuracy of Equation (1). However, in fact, even if there exists parameter uncertainty *w*, the output of the closed-loop system is able to track F(t) asymptotically and robustly, provided that Equation (17) can remain stable in the presence of *w*. Therefore, the tracking error *e* is able to converge to zero robustly by selecting $K_{1}$ and $K_{2}$ appropriately.

Following, an introduction of the selection of $K_{1}$ and $K_{2}$ is provided. For this issue, there are some classic ways, such as pole placement, linear quadratic regulator (LQR), Lyapunov method, and so on. In this paper, we design $K_{1}$ and $K_{2}$ by applying the LQR approach. In the following, we make a simple description of the LQR approach.

Firstly, ignoring the reference, we rewrite Equation (17) as follows:

(19) x˙z˙=A0G2CG1xz+B0u=A¯X+b¯u u=KX=K1x+K2zK=K1K2 y=C0xz=c¯X V=gX+hu

where *V* represents the controlled output that one would like to make as small as possible in a shorter time in the framework of LQR.

In the most general form of the LQR method, the standard LQR object function is defined as:

(20) $$J_{LQR1}=\int_{0}^{\propto }\left(X^{T}QX+u^{T}Ru\right)dt$$

The goal of the LQR method is to minimize Equation (20). However, choosing *Q* and *R* to minimize Equation (20) is a challenging task. To tackle this dilemma, we will figure out the two matrices through an indirect way.

For this purpose, choose the following kind of LQR object function:

(21) $$J_{LQR2}=\int_{0}^{\propto }\left({∥V(t)∥}^{2}+\rho {∥u(t)∥}^{2}\right)dt$$

Selecting the controlled output:

(22) $$V\left(t\right)=\left[\begin{array}{c} y(t)\\ \gamma \dot{y}\left(t\right)\\ \beta z_{1}\left(t\right) \end{array}\right]$$

we have:

(23) $$g=\left[\begin{array}{c} \bar{c}\\ \gamma \bar{c}\bar{A}\\ c_{1} \end{array}\right],\;h=\left[\begin{array}{c} 0\\ \gamma \bar{c}\bar{b}\\ 0 \end{array}\right],\;c_{1}=\left[\begin{array}{ccc} 0 & 0 & \beta \;0 \end{array}\right]$$

Since V=gX+hu, the criterion in Equation (21) is a special form of the criterion Equation (20) with:

(24) $$Q=g^{T}g,R=h^{T}h+\rho I$$

Then, the feedback gain can be calculated by:

(25) $$K=R^{-1}{\bar{b}}^{T}\bar{P}$$

where the matrix $\bar{P}$ is the solution of the following algebra Riccati equation:

(26) $${\bar{A}}^{T}\bar{P}+\bar{P}\bar{A}+Q-\bar{P}\bar{b}R^{-1}{\bar{b}}^{T}\bar{P}=0$$

Clearly, we can get suitable $K_{1}$ and $K_{2}$ by tuning the three above parameters in the LQR framework.

**Remark** **1.** *In this paper, the reference we select is the sinusoidal reference because our application background focus is scanning and imaging. However, not only is the above control design methodology able to solve the specific tracking control problem in this paper, but it is also able to extend to wider application background. Although F(t) will be changed in the other different application backgrounds, the tracking control problem can still be tackled robustly by following the above control design framework, and the only difference is to change Equation (18) into the corresponding internal model according to the form of Equation (13), which is the dynamic structure of F(t). Considering that Equation (13) can generate a large class of functions [23], obviously, the above control design framework provides a kind of method to force the MEMS device to track different kinds of references robustly, which is another goal of this paper.*

## 4. Simulation and Comparison

This section is organized as follows. Firstly, a simple process of the controller parameters’ tuning is given, and the tracking performance, as well as the robustness examination of the proposed controller are provided. Secondly, a comparison between the proposed controller and the PID controller is shown, and some brief analysis is given. Note that the sinusoidal wave scanning trajectory with the amplitude of one is chosen, i.e., F(t) = sin (σt).

### 4.1. Simulation Results of The Proposed Controller

**Figure 3:**
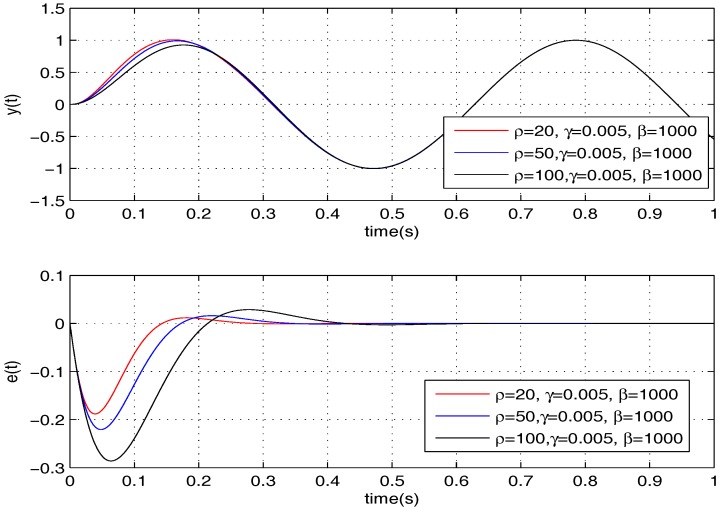
Tuning of ρ.

**Figure 4:**
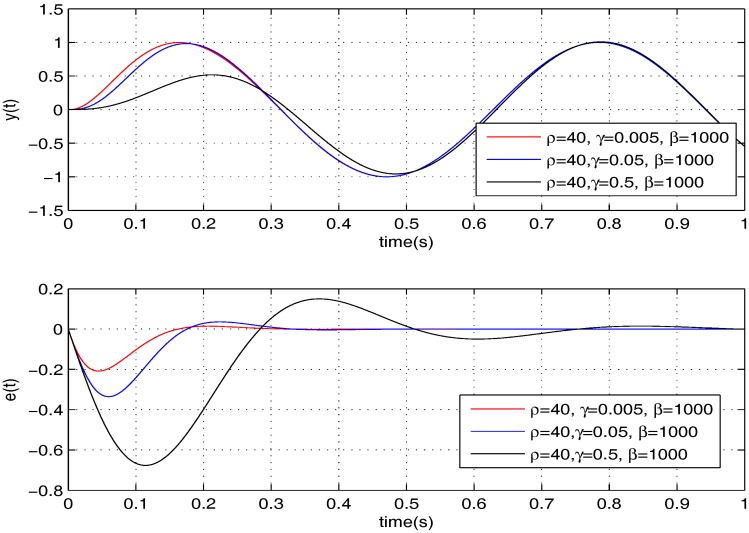
Tuning of γ.

**Figure 5:**
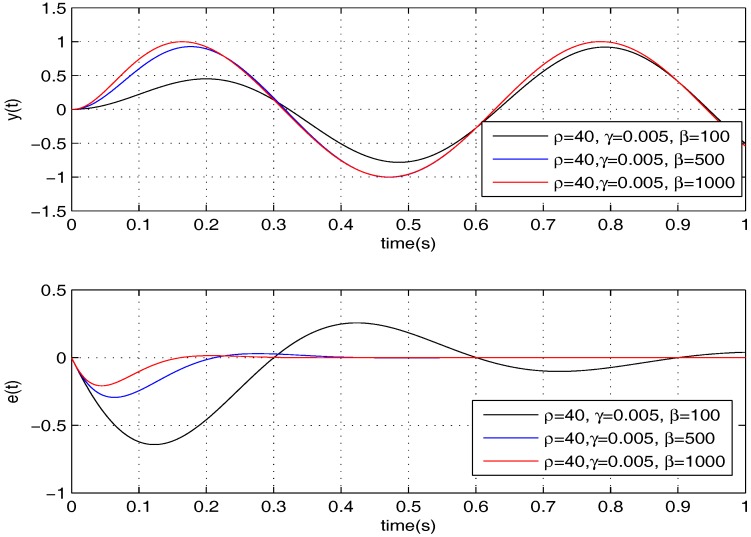
Tuning of β.

Firstly, we need to tune the parameters of the proposed controller according to the specific micromirror presented in this paper. Thus, we do simulations to learn how every parameter influences the tracking performance. Following, for convenience, we take σ=10 as an example. The results are shown in Figure 3, Figure 4 and Figure 5.

Based on the trends shown in the above three figures, we select ρ=10, γ=0.001, β=2000, and we further reach $K_{1}=\left[-0.094,-1.4\times 10^{-4}\right]$ and $K_{2}=\left[200,6.7\right]$.

**Figure 6:**
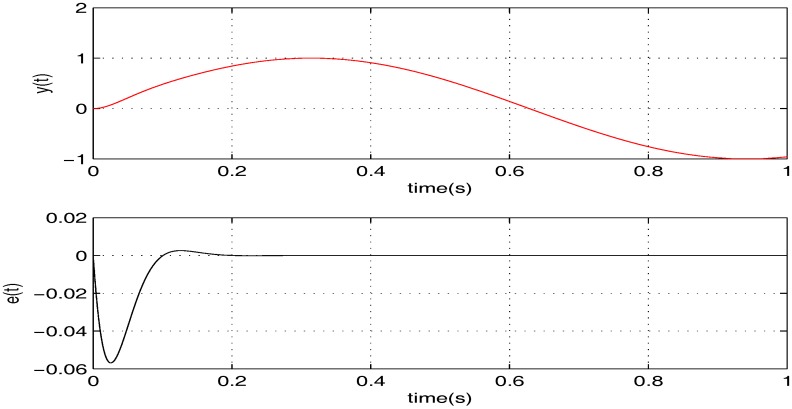
The tracking performance of the proposed controller when σ=5.

**Figure 7:**
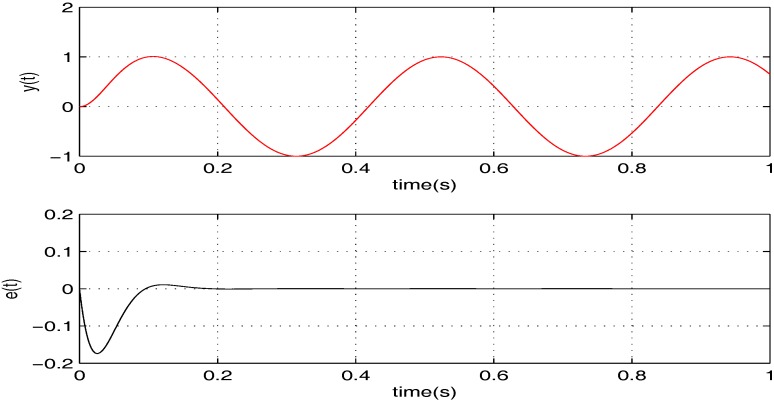
The tracking performance of the proposed controller when σ=15.

To illustrate the tracking performance, the proposed controller is verified for two cases, namely: (1) σ=5; (2) σ=15. The simulation results for the two cases are shown in Figure 6 and Figure 7, in which the output responses of the closed-loop system and the tracking errors are given. Obviously, the outputs track the given references precisely in both cases with respect to frequency variations.

**Figure 8:**
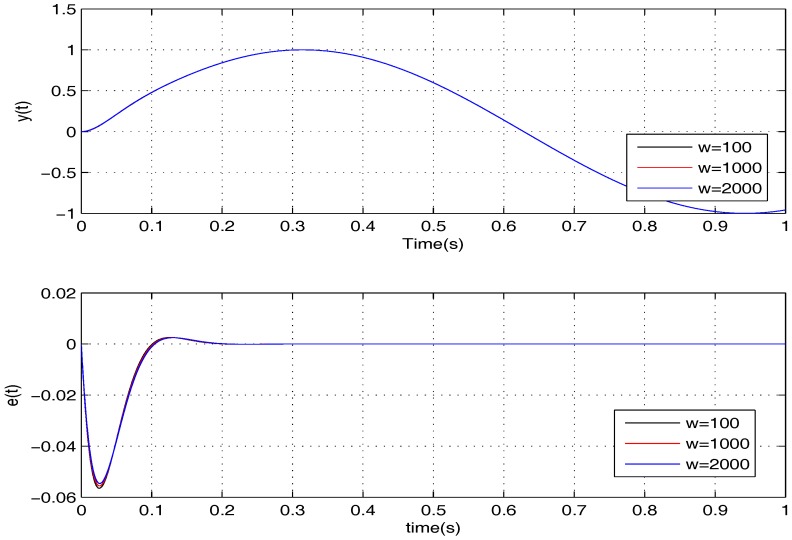
The performances of the proposed controller under *w* when σ=5.

**Figure 9:**
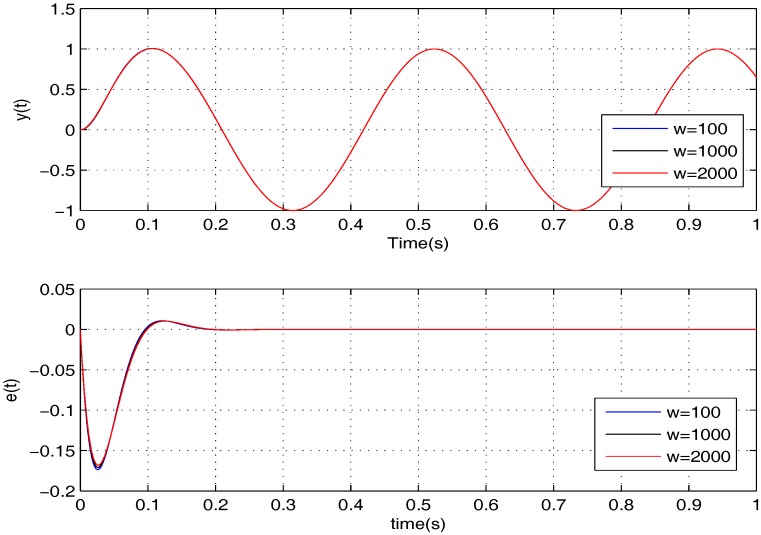
The performances of the proposed controller under *w* when σ=15.

To further examine the robustness provided by the proposed controller, it is verified for two cases, namely: (1) σ=5,w=100,1000,2000; (2) σ=15,w=100,1000,2000. The simulation results of the two cases are shown in Figure 8 and Figure 9. Apparently, the tracking error *e* can converge to zero in all of the cases in the presence of *w*, meaning that the uncertainties are handled nicely by the proposed controller.

In summary, through the simulation results in this subsection, we can conclude that the proposed controller is able to provide outstanding tracking performance robustly.

### 4.2. Comparison with PID Controller

In a great amount of application scenarios, the well-known PID controller is applied to control the given object. In this subsection, we are going to make a comparison between the proposed controller and the PID controller. The form of PID controller is shown as follows. In this paper, we select $K_{i}=10$, $K_{p}=1$, $K_{d}=0.0001$.

(27) $$\begin{array}{c} u_{PID}\left(t\right)=K_{i}\int_{0}^{t}e\left(\rho \right)d\rho +K_{p}e\left(t\right)+K_{d}\frac{de(t)}{dt} \end{array}$$

**Figure 10:**
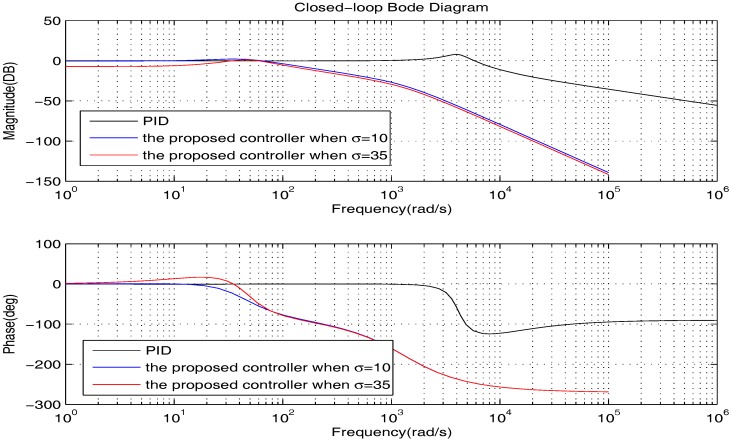
The bode diagrams comparison of the two controllers.

We firstly show the closed-loop bode diagrams under the two controllers in Figure 10 to compare the dynamic characteristics. From Figure 10, we can obtain that: (1) the closed-loop system under the PID controller has a much wider bandwidth and larger resonance peak; (2) the closed-loop systems under the proposed controller have a much greater attenuation rate at a high frequency range. Thus, we can summarize that: (1) the closed-loop system under the PID controller has shorter response time; (2) the closed-loop systems under the proposed controller can deal with the high frequency noise better.

**Figure 11:**
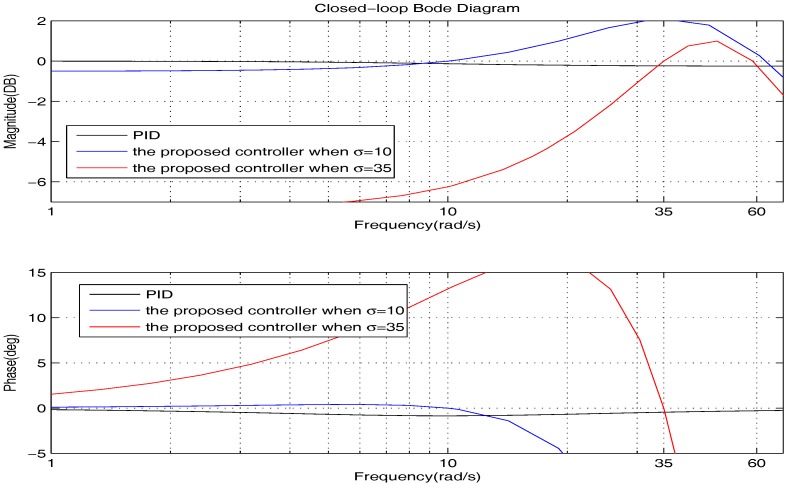
The bode diagrams comparison of the two controllers at low frequency.

Usually, σ is in the low frequency range of the bode diagrams. Therefore, to investigate the tracking performance synthetically, we further plot the low frequency range of the bode diagrams clearly in Figure 11. From Figure 11, we can obtain that: (1) the closed-loop systems under the proposed controller can track their given reference with zero tracking errors; (2) the PID controller is able to provide good tracking accuracy when σ is small, but the steady state error is increasing with the growth of σ. To get deeper impressions, in the following, we will illustrate the tracking performance comparison for some specific cases.

**Figure 12:**
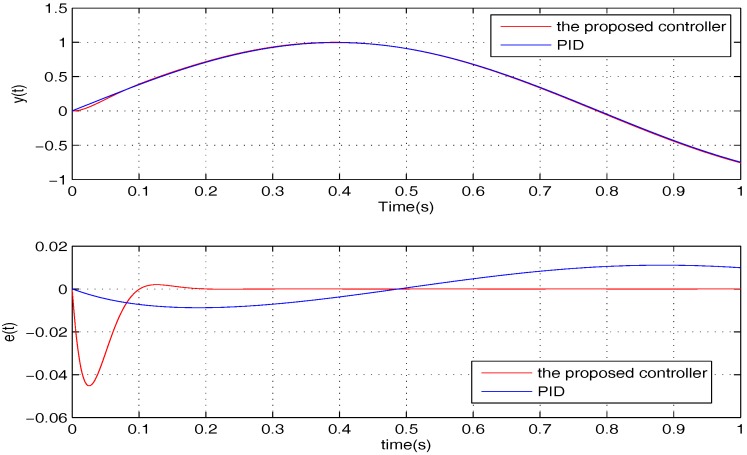
The performances comparison of the two controllers when σ=4.

**Figure 13:**
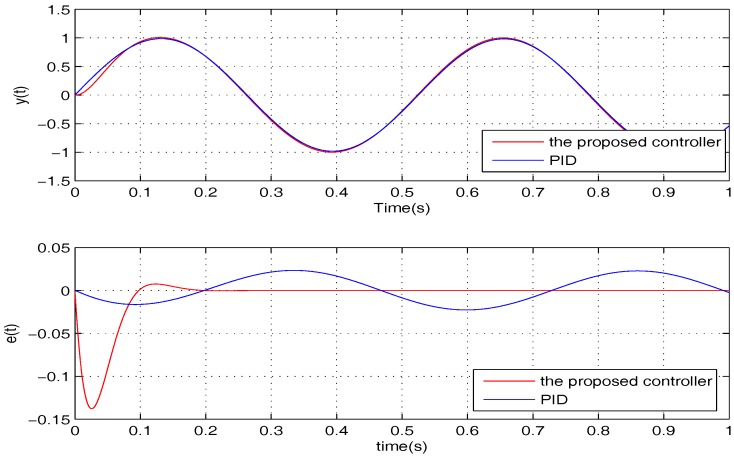
The performances comparison of the two controllers when σ=12.

**Figure 14:**
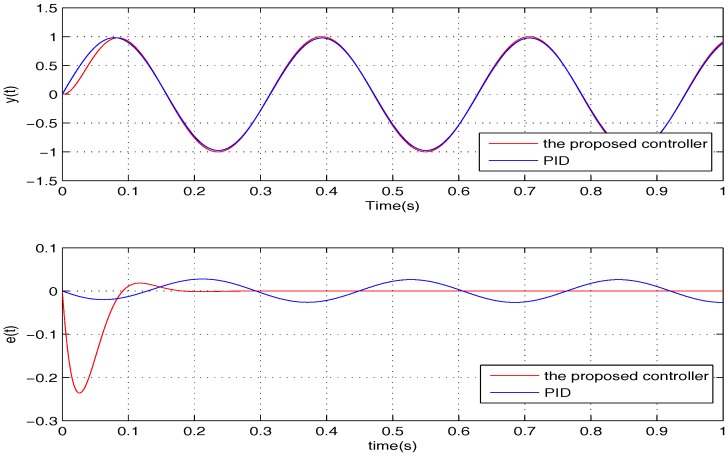
The performances comparison of the two controllers when σ=20.

**Figure 15:**
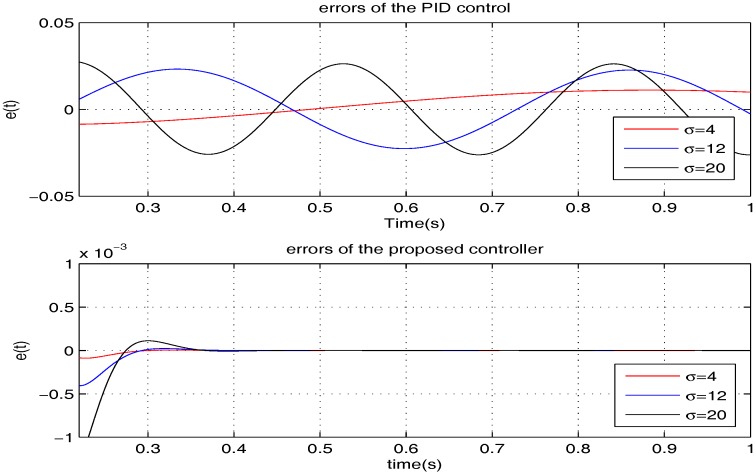
The errors comparison of the two controllers.

To compare the tracking performance, the two controllers are verified for three specific cases, namely: (1) σ=4; (2) σ=12; (3) σ=20. The simulation comparison results are shown in Figure 12, Figure 13 and Figure 14, and the steady state errors comparison is shown in Figure 15. It is clear that: (1) the PID controller provides a quicker response; (2) the proposed controller provides better accuracy.

As we have mentioned above, the accuracy of the PID controller is becoming worse with the growth of σ, which brings an issue worthy of discussion. The analyses are shown as follows. Firstly, we need to transform the internal model in Equation (15) into the form of the transfer function, which is shown as follows:

(28) $$G_{z}\left(s\right)=\frac{as+b}{s^{2}+\sigma^{2}}$$

Clearly, the structure characteristic of Equation (28) is $\left(s^{2}+\sigma^{2}\right)$. It is generally known that the transfer function of the PID controller is:

(29) $$\begin{array}{c} G_{PID}\left(s\right)=K_{i}s^{-1}+K_{p}+K_{d}s \end{array}$$

Actually, just focusing on the integral term, Equation (29) is a special case of Equation (28) when σ=b=0 in the aspect of classic control theory. According to [20], we can learn that the tracking accuracy of Equation (15) relies heavily on the accuracy of σ. Clearly, when σ is sufficiently small, which approaches zero closely, the integral term may be able to be an approximation of Equation (28) in the actual application. However, as the number of σ becomes larger, the approximation effect of the integral term becomes worse. Up to some level, the integral term cannot act as a compensator like the internal model any more, leading to terrible steady state performances. Through these analyses, it is not hard to understand the above phenomenon shown in the simulation results.

**Figure 16:**
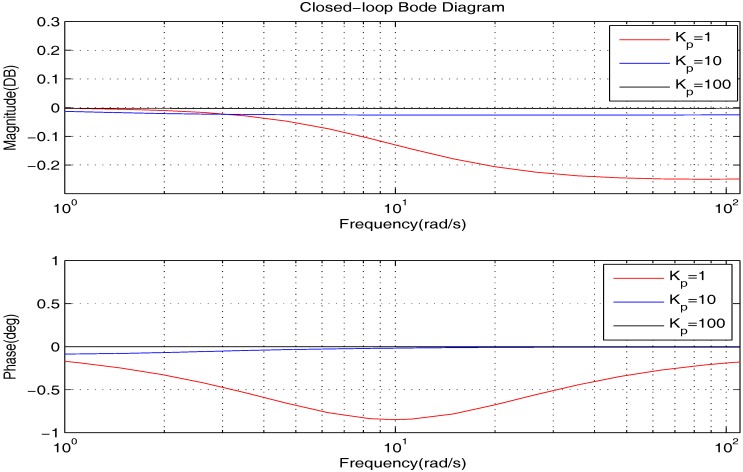
The bode diagrams comparison of the PID controller with different $K_{p}$ at low frequency.

**Figure 17:**
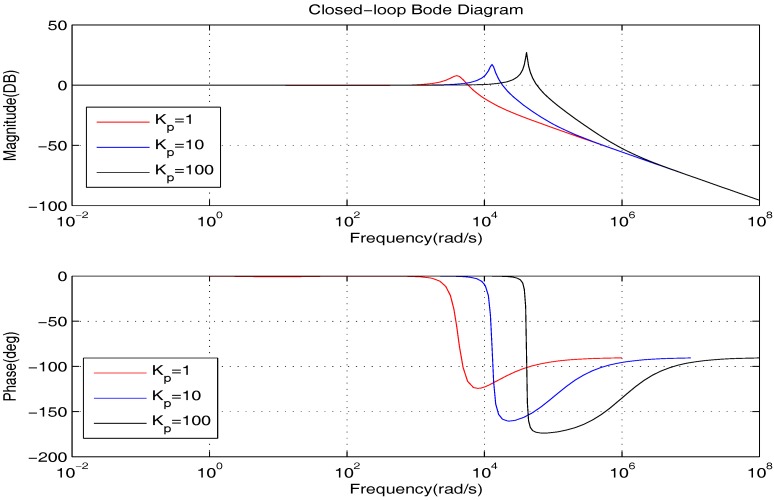
The bode diagrams comparison of the PID controller with different $K_{p}$.

In the framework of PID control, it is able to decrease the steady state error by increasing $K_{p}$, which is as shown in Figure 16. However, from Figure 17, we learn that such a method will lead to a wider bandwidth and a larger resonance peak. On this occasion, the high-frequency noise will be amplified, which is greatly against the actual application. Therefore, to obtain better precision, the proposed controller is a much better choice.

In addition to accuracy, we are going to compare the robustness of the two controllers. The robustness of the PID control is verified for two cases, namely: (1) σ=5,w=100,1000,2000; (2) σ=15, w=100,1000,2000. The simulation results for these cases are shown in Table 4. From Table 4, we learn that the tracking error *e* cannot converge when w=1000,2000, meaning that the uncertainties are not able to be handled nicely by the PID controller compared with the proposed controller. Apparently, the proposed controller can provide much better robustness.

Above all, in the current application background, the proposed controller is a better option compared with PID control.

## 5. Experimental Setup and Results

**Figure 18:**
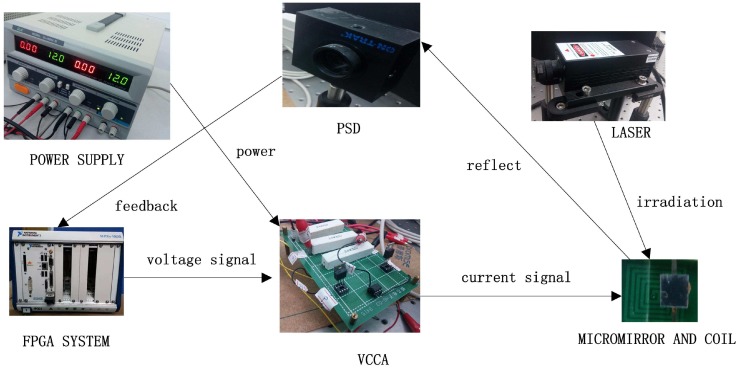
The composition of the platform.

**Figure 19:**
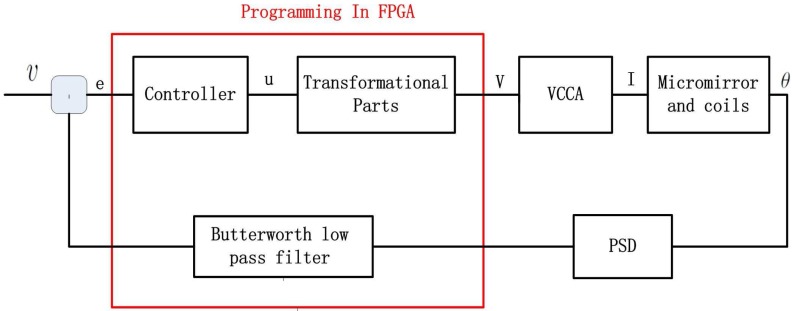
Schematic of the experimental setup.

First of all, a brief introduction of the experimental system is provided, and the structure is shown in Figure 18. The system consists of a He-Ne laser, a micromirror together with coils, a PSM2-10 position sensing detector (PSD), a voltage-controlled current amplifier (VCCA) circuit, an NI PXI-7852R field programmable gate array (FPGA) card and some additional optical device. The deflection of the micromirror will result in the change of the position of the light spot reflected on the PSD screen, and the positional signals from PSD can be received by the FPGA card. The proposed feedback control algorithm has been programmed, compiled and downloaded into the FPGA card in advance in a LabVIEW environment. The signal of the controller produced by the FPGA card is in the form of voltage. The voltage signal is converted to current by the VCCA circuit proportionally, and the ratio is one to one. Thus, the planar coils under the micromirror can be powered, generating the torque to result in the angular deflection. The schematic of the experimental setup is shown in Figure 19, where *V* denotes the control output generated by FPGA and *I* denotes the output current of VCCA. Note that, in order to eliminate the influence of sensing noise, we will apply an extra Butterworth low pass filter.

**Figure 20:**
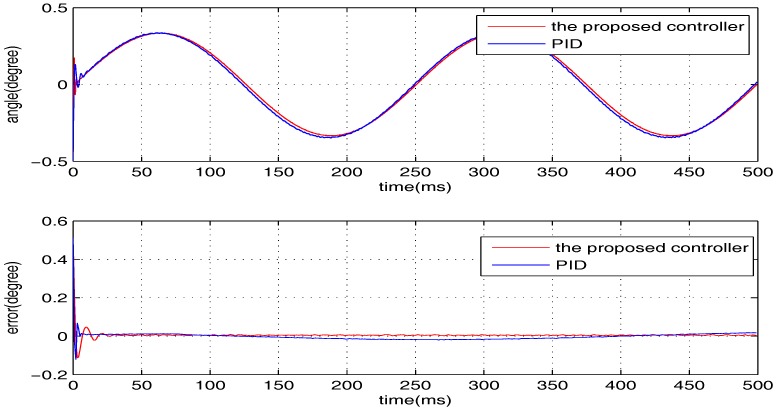
The performance of two controllers when σ=2π×4.

**Figure 21:**
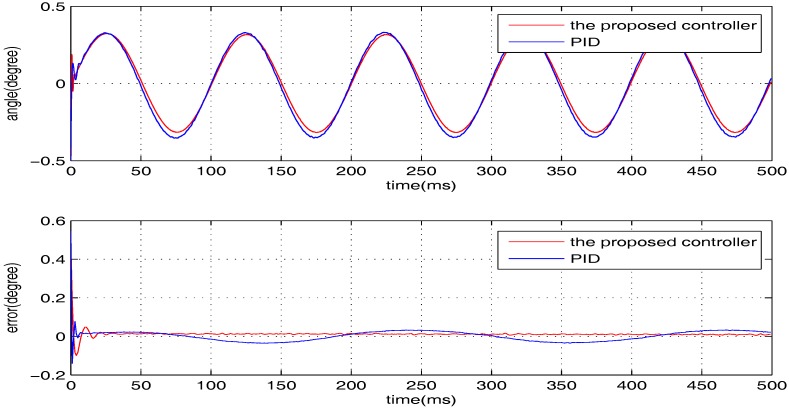
The performance of two controllers when σ=2π×10.

**Figure 22:**
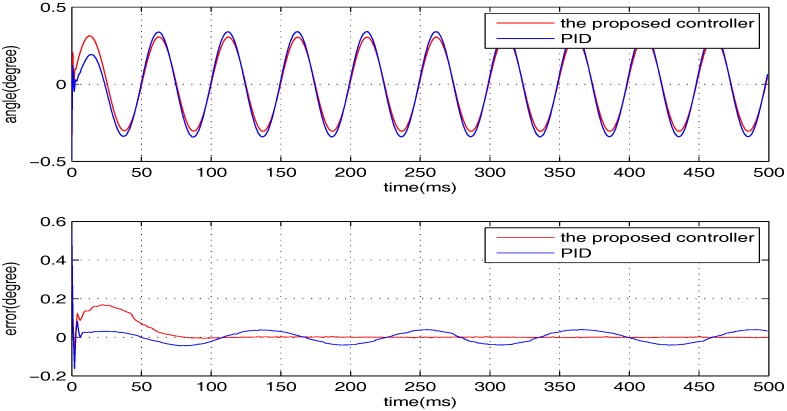
The performance of two controllers when σ=2π×20.

**Figure 23:**
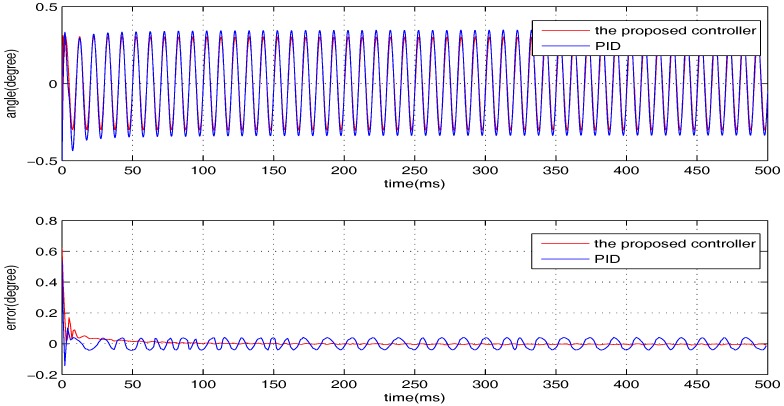
The performance of two controllers when σ=2π×100.

The sinusoidal wave scanning trajectory with the amplitude 0.006 rad (0.34 degrees) is chosen, i.e., F(t)=0.006sin (σt). In the experimental illustration, the proposed controller is verified for four cases, that is: (1) σ=2π×4; (2) σ=2π×10; (3) σ=2π×20; (4) σ=2π×100. The experimental results for the four cases are shown in Figure 20, Figure 21, Figure 22 and Figure 23, in which the output responses of the closed-loop system and the tracking errors are given. It can be observed that the tracking performances of all three cases are remarkably favourable with respect to frequency variations. In general, we can obtain that the effectiveness of the internal model-based controller is manifest, which is able to satisfy the actual demand of scanning or imaging application.

**Figure 24:**
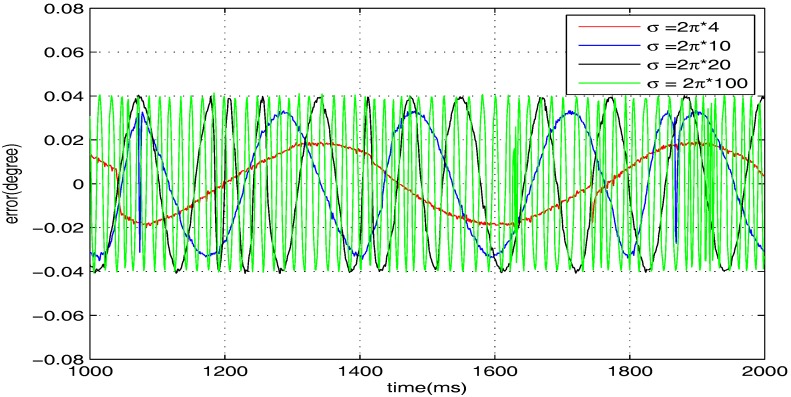
Steady state errors of the PID controller.

**Figure 25:**
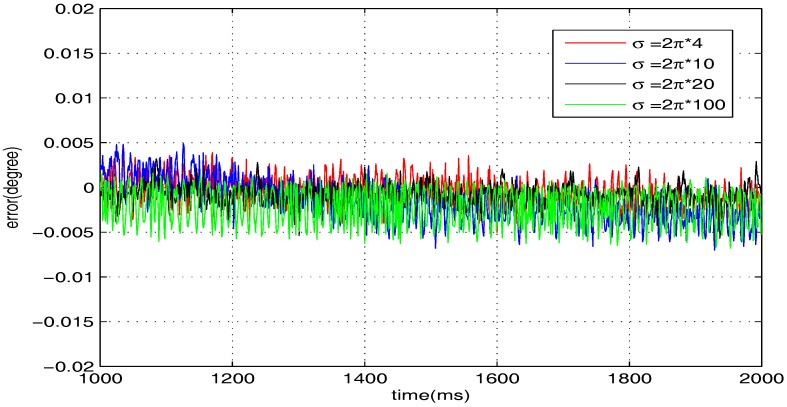
Steady state errors of the internal model-based controller.

In contrast, we implement a well-known proportion-integral-derivative (PID) controller and test it for the above four cases. The effectiveness of the PID controller is also shown in Figure 20, Figure 21, Figure 22 and Figure 23. Clearly, the outputs of the closed-loop system under PID control converge to the given references faster, but there exists distinct steady state errors in the cases of σ=2π×10, σ=2π×20 and σ=2π×100. To obtain deeper impressions, we further illustrate the steady state errors of the two controllers in all of the cases, which are shown in Figure 24 and Figure 25. It is obvious that the steady state performances of the internal model-based controller are independent of σ, which are favourable in all four cases. It is remarkable that the steady state errors of the PID controller increase with σ, which is consistent with the analyses in Section 4. In general, we can ulteriorly conclude that the internal model-based controller is capable of providing outstanding tracking performances.

## 6. Conclusions

In this paper, an internal model-based controller is introduced to solve the angular tracking control problem for an MEMS micromirror. With the application of the internal model principle, the controller is able to make the output of the closed-loop system track the desired reference asymptotically and robustly in the presence of uncertainty. The effectiveness of the approach is verified by experimental results, and clearly, the performances are satisfying. Such a design methodology can be further applied to other MEMS devices.

## Figures and Tables

**Table 1:** Parameters of the micromirror.

Symbol	Parameter	Value
$W_{beam}$	width of the torsion bar	250 μm
$L_{beam}$	length of the torsion bar	2 mm
$t_{beam}$	thickness of the torsion bar	250 μm
$\rho_{beam}$	density of the torsion bar	970 kg/m${}^{3}$
*E*	modulus of the torsion bar	0.75 MPa
$L_{m}$	length of the mirror	4 mm
$W_{m}$	width of the mirror	2 mm
$t_{m}$	thickness of the mirror	250 μm
$\rho_{m}$	density of the mirror	1670 kg/m${}^{3}$

**Table 2:** Microcoils’ parameters.

Parameter	Value
Trace width	250 μm
Spacing	250 μm
Turns	10 turns
Cu thickness	35.56 μm
Resistance	0.033 Ω (at 20 degrees Celsius)

**Table 3:** FEA simulation result.

*I* (A)	*u* (N·m)
0.1	0.06257 × $10^{-7}$
0.2	0.1258 × $10^{-7}$
0.3	0.1903 × $10^{-7}$
0.4	0.2552 × $10^{-7}$
0.5	0.3216 × $10^{-7}$
0.6	0.3889 × $10^{-7}$
0.7	0.4564 × $10^{-7}$
0.8	0.5256 × $10^{-7}$
0.9	0.6220 × $10^{-7}$

**Table 4:** Performance of the PID control under *w*.

	w=100	w=1000	w=2000
σ=5	1.1% steady state error	Unstable	Unstable
σ=15	2.4% steady state error	Unstable	Unstable
